# Field size as a determinant of common vole population density

**DOI:** 10.1002/ps.70410

**Published:** 2025-11-28

**Authors:** Emil Tkadlec, Radek Aulický, Marek Bednář, Marcela Fraňková, Eva Jánová, Václav Stejskal, Jan Šipoš, Josef Suchomel

**Affiliations:** ^1^ Department of Ecology and Environmental Sciences, Faculty of Science Palacký University Olomouc Olomouc Czech Republic; ^2^ Czech Agrifood Research Center Prague Czech Republic; ^3^ Department of Forest Ecology, Faculty of Forestry Mendel University in Brno Brno Czech Republic; ^4^ Department of Zoology, Fisheries, Hydrobiology and Apiculture, Faculty of AgriSciences Mendel University in Brno Brno Czech Republic

**Keywords:** alfalfa, density–area relationship, forage crops, landscape heterogeneity, *Microtus arvalis*, rodent pest management

## Abstract

**BACKGROUND:**

Environmental heterogeneity in agricultural landscapes is a key driver of biodiversity and ecological processes, yet its role in shaping the population dynamics of pest species remains insufficiently studied. In central Europe, post‐war collectivisation led to widespread homogenisation of farmland, notably through the enlargement of arable fields. This study examined the effects of this structural simplification by assessing the relationship between field size, a key configurational component of landscape heterogeneity in farmland, and the abundance of the common vole (*Microtus arvalis*), a major agricultural pest, monitored in forage fields over 7 years in the Czech Republic.

**RESULTS:**

Linear mixed models, accounting for season, crop type and altitude, revealed a robust, nonlinear positive relationship between field size and vole population density, with the most pronounced effect in fields smaller than 20 ha. Alfalfa fields consistently supported the highest vole densities in autumn, indicating that both habitat quality and patch size jointly influence vole abundance.

**CONCLUSION:**

This study provides the first empirical evidence of a positive density–area relationship in voles, challenging theoretical expectations and previous field evidence predicting negative or neutral responses in ground‐dwelling species. The findings have important implications for nationwide pest management and landscape planning, suggesting that reducing field size could help mitigate crop damage while promoting more resilient and ecologically balanced agroecosystems. © 2025 The Author(s). *Pest Management Science* published by John Wiley & Sons Ltd on behalf of Society of Chemical Industry.

## Graphical abstract

Population densities of the common vole, an agricultural pest, increase nonlinearly with forage field size, especially in fields below 20 ha. Reducing the field size may help limit crop damage in farmland.

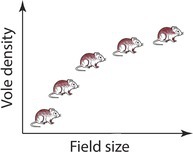

## 1 INTRODUCTION

Environmental heterogeneity is crucial in agrarian landscapes, affecting various aspects of agriculture and its sustainability. It fosters biodiversity, supports ecosystem services and provides opportunities for sustainable farming practices, ultimately contributing to food security and the well‐being of rural and urban populations.^1^, ^2^, ^3^ Agriculture intensification, one of the main drivers of the biodiversity loss,^4^ entails several interrelated changes, including the loss of landscape elements, increased use of fertiliser and pesticides, and the enlargement of farms and fields, often in response to the introduction of modern machinery. Many central European countries, including the Czech Republic, have undergone profound transformations of their agrarian landscape, marked by a significant reduction in habitat heterogeneity. Following the collectivisation of agriculture after World War II, extensive field consolidation led to a coarse‐grained, homogeneous landscape. This landscape simplification, characterised by the enlargement of arable fields and the removal of semi‐natural features such as hedgerows, small woodlots and grass strips, has had far‐reaching ecological consequences, including steep declines in biodiversity across multiple taxa.^5^, ^6^, ^7^

Landscape structure, often used as a proxy for environmental heterogeneity, is typically described in terms of its compositional component, such as diversity and abundance of habitat types, and its configurational component, such as field size and edge density.^8^ Among these, field size plays a central role in shaping biodiversity and ecological interactions, including predator–prey dynamics, dispersal processes and resource availability.^2^, ^4^, ^9^, ^10^, ^11^ Although the effects of landscape configuration on biodiversity have been widely studied in birds, insects and plants, its implications for pest species abundance are less well understood. This uncertainty stems partly from species‐specific dispersal behaviours, especially specific movement abilities and orientation toward the resource patch, which can affect the relationship between patch size and population density.^12^ A meta‐analysis has demonstrated that resident insect or mammalian species specialised for living within the patch rather than at its edge are more prone to exhibit the positive relationship.^13^ Although the total number of immigrants per patch typically increases with patch size, the effect of patch size on population density can be positive, negative or neutral, depending on a combination of behavioural and ecological traits. For instance, most rodent species show no relationship or even a negative one between patch area and population density.^14^

Understanding what determines species abundance, and how these abundances are regulated, has long been central to population ecology theory, dating back to foundational work by Nicholson^15^ and Andrewartha and Birch.^16^ At broad spatial scales, it is often assumed that species are most abundant at the centre of their geographic ranges.^17^ At local scales, abundances should reflect how well a site meets the species’ ecological requirements, including dynamic population processes over time.^18^ Resource availability, competition, predation and disease risk, and other environmental factors, such as climatic influences and soil characteristics, are major drivers of spatial variation in abundance. Although less frequently studied in voles, landscape heterogeneity has been linked to variation in population dynamics among small rodents, with more homogeneous landscapes promoting higher vole population densities and more pronounced population cycles.^19^, ^20^, ^21^ However, this hypothesis remains difficult to test rigorously.

The common vole is one of the most abundant rodent species in central European farmlands and is known for recurrent population outbreaks, which can cause significant crop damage.^22^, ^23^ Understanding how landscape structure influences vole populations is therefore of both ecological and economic importance. Despite this, empirical studies quantifying the relationship between field size and vole abundance remain scarce. This study aims to address this gap by assessing the effect of field size – a dominant landscape component influencing biodiversity in farmland – on the abundance of the common vole in their preferred crops. By directly examining the relationship between vole population density and field size, we provide a means of quantitatively testing the ecological hypothesis that larger fields in homogeneous landscapes promote higher vole densities. Furthermore, by linking a spatial metric of landscape structure with field survey data, we assess whether reducing field size could help manage vole populations and contribute to more ecologically balanced and sustainable agroecosystems.

## 2 MATERIALS AND METHODS

### 2.1 The common vole

The common vole is a major agricultural mammal pest in Europe, capable of reaching population densities exceeding 2000 individuals per hectare,^24^ which is substantially higher than other vole species and comparable only with the house mouse in Australia.^25^ It is a small‐bodied vole that inhabits burrow systems of varying complexity and prefers open grassy habitats with herbaceous vegetation, such as meadows, field margins, pastures, orchards, gardens, ditches, road verges or even grassy forest clearings and nurseries. In farmland, it favours forage crops, such as alfalfa or clover, but it also thrives in winter cereals and oilseed rape. The population dynamics of the common vole are characterised by periodic large‐scale outbreaks every 2–5 years,^26^ although the regularity of these cycles varies among regions.

### 2.2 Field data

**Figure 1:**
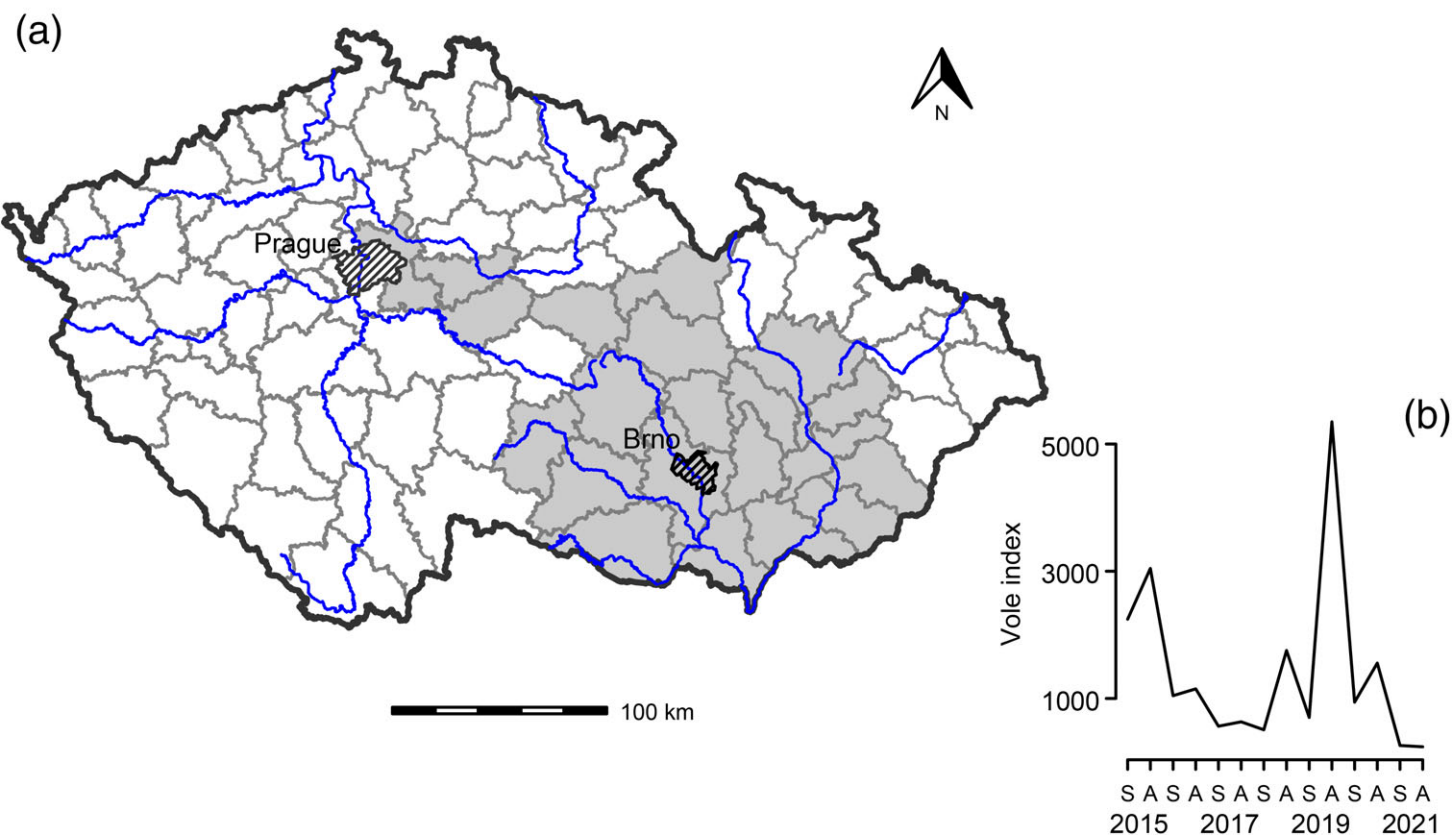
(a) Map of the Czech Republic showing the 22 sampled districts (shaded) between 2015 and 2021. (b) Seasonal population dynamics of the common vole during the study period (S, spring; A, autumn). The vole index is based on the number of active burrow entrances per hectare.

Between 2015 and 2021, we collected data in 22 of the 77 territory districts of the Czech Republic (average area ~1000 km^2^) (Fig. 1(a)), obtaining 7–15 estimates per district and season. Most districts were in lowland areas with intensive agricultural production, primarily focused on cereals and oilseed rape. Alfalfa is the main forage crop for livestock, but because of a long‐term decline in cattle numbers, forage fields now exist as small, scattered patches across the agrarian landscape. At elevations above 400 m a.s.l., alfalfa is typically replaced by red clover. Both alfalfa and clover are usually harvested and transported directly as fresh fodder to cattle sheds. All sampled districts had similar field size structure, with lowland field sizes tending to increase (linear regression, *P* = 0.07).

Each season, we sampled more than 200 forage crop fields (including alfalfa, alfalfa–grass mixtures and clover) of varying sizes, conducting surveys twice a year, in spring (March–April) and autumn (October–November). Vole density was estimated using the burrow population index, based on counts of active burrow entrances per hectare. Entrances were recorded along four transects, each 100 m long and 2.5 m wide, and the total count was multiplied by 10 to obtain a per‐hectare estimate. The index is slightly nonlinear, tending to overestimate population size at higher densities.^27^ Field size was measured using the OneSoil web application (https://onesoil.ai), based on satellite imagery, with a precision of 0.1 ha. We did not record crop types surrounding each forage field or measure distances to neighbouring forage fields to assess patch isolation. However, most fields are connected by a network of grassy verges and roadside ditches, which provide suitable overwintering habitats and dispersal corridors for voles. For this reason, patch isolation was not included in our analysis.

### 2.3 Statistical analysis

Over the 7‐year study, we obtained 3262 paired measurements of vole population density and field size. After excluding under‐represented crop types and grassy habitats, which do not represent ‘true patches’ in the landscape, 2531 observations remained for statistical analysis. To stabilise variance, the vole index was transformed using the Box–Cox function (lambda = 0.05). Zero values were replaced with 5, corresponding to half of the minimum detectable estimate (10) using our method. We analysed variation in the vole index using linear mixed models (LMMs), with Gaussian error distributions. Fixed effects included field size (mean = 17.8 ha, range = 0.4–117 ha), and three confounding covariates: season (spring, *n* = 1277; autumn, *n* = 1254), crop type (alfalfa, *n* = 2136; alfalfa–grass mixture, *n* = 173; clover, *n* = 222), and altitude (range = 159–683 m). Field size was log‐transformed to linearise its relationship with vole density. We included hierarchically nested random effects of crop within district within season and year (1|year/season/district/crop) on the intercept to account for non‐independence of data and local deviations from the mean. Because many fields were surveyed repeatedly, we also included field identity (*n* = 741) as a random intercept to account for repeated measurements. A set of candidate models was constructed using different combinations of four fixed effects and their interactions. Model selection was based on the Akaike Information Criterion corrected for small samples (AICc). A model was considered well‐supported if its AICc was at least 2 units lower than competing models. We also calculated marginal and conditional pseudo‐coefficients of determination, which indicate the proportion of variance explained by fixed effects only and the total proportion of variance explained by both fixed and random effects.^28^ Model predictions were back‐transformed to the original scale and corrected for bias using the delta method. All analyses were conducted in R^29^ using the lme4 package,^30^ which provides tools for fitting and evaluating LMMs.

## 3 RESULTS

Common vole numbers fluctuated substantially during the studied period, with seasonal means ranging from 268.8 entrances per hectare in autumn 2021 to 6325.9 in autumn 2019 (Fig. 1(b)). The overall mean population density of the common vole was 1612 (*SD* 3007) active burrow entrances per hectare. As expected, vole densities were generally higher in autumn than in spring (spring: mean = 974.2, *SD* = 1730.2; autumn: mean = 2262.5, *SD* = 3790.4).

**Figure 2:**
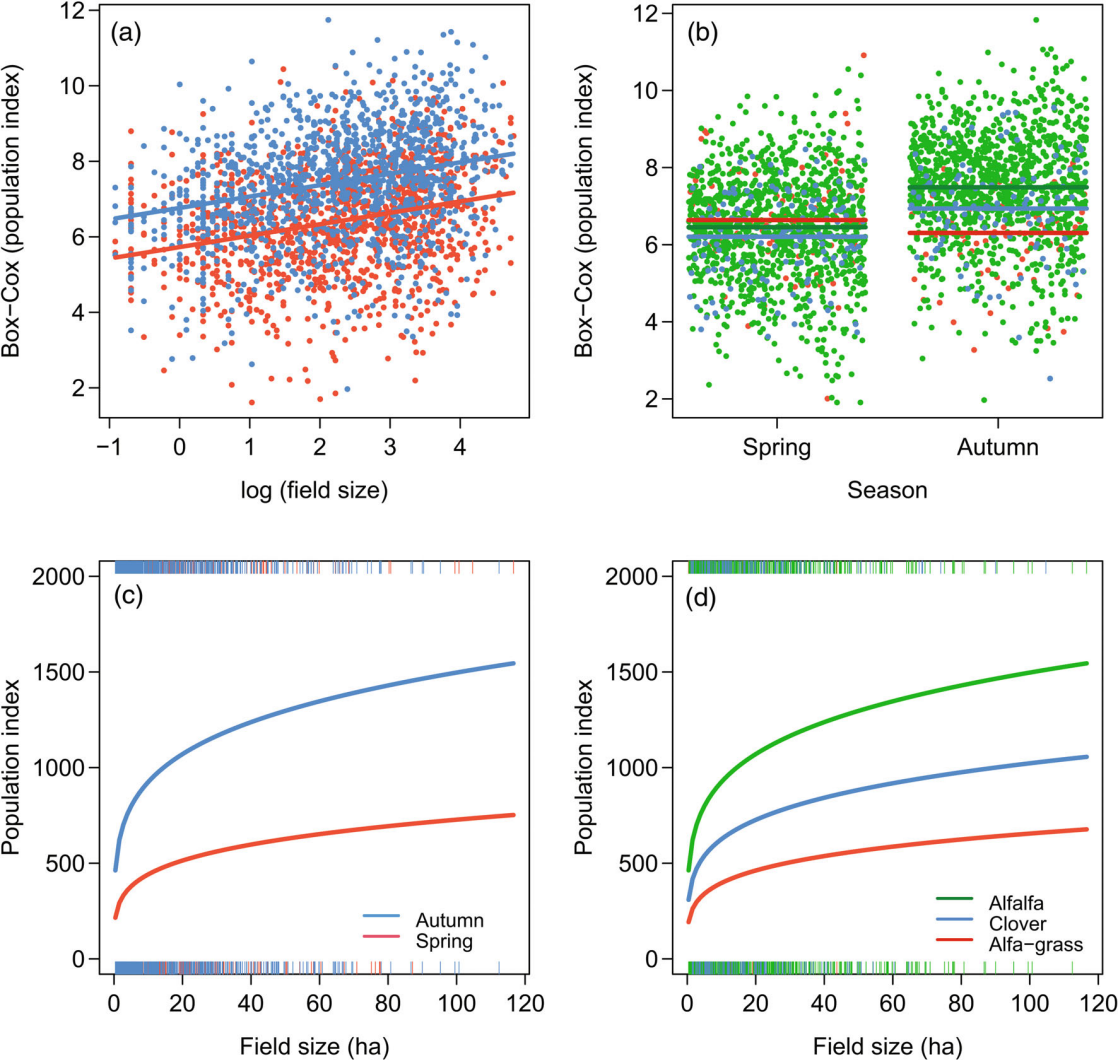
(a) Linear regression of vole population density on field size in spring and autumn, as predicted by model 6 for alfalfa. Vole densities were Box–Cox transformed, field sizes were log‐transformed. (b) Seasonal vole population densities across crop types. (c) Regression of vole population density on field size in spring and autumn, back‐transformed to the original scale. (d) Regression of autumn vole population density on field size across crop types, back‐transformed to the original scale.

The statistical modelling yielded three key findings. First, all LMMs (Table 1) revealed a consistent positive effect of log field size on population density. The estimated regression coefficients were robust, ranging from 0.298 (*SE* = 0.051) in model 4 to 0.309 (*SE* = 0.051) in models 2 and 3. Four models received strong support based on AICc. Model 6 had the lowest AICc, but models 5, 7 and 9 had ΔAICc <2, indicating equivalent support. All four models included field size, season, crop and the interaction between season and crop. Models 6 and 7 also included altitude, suggesting that vole densities may additionally be influenced by altitude. Model 6 predicted a linear increase in transformed vole density with increasing log field size (Fig. 2(a)), with parallel slopes across crop types. However, the regression was noisy, and the proportion of explained variance was low, with marginal and conditional *R* ^2^ values of 0.057 and 0.65, respectively. Back‐transformation to the original scale revealed a strongly nonlinear relationship, with the steepest increases in fields smaller than 20 ha (Fig. 2(c), (d)). Second, including season, crop type and their interaction in model 4 substantially improved the model fit, reducing the AICc by 12.3 units. This suggests that vole densities differ among crops depending on season, with alfalfa supporting the highest autumnal densities, followed by clover, and alfalfa–grass mixtures supporting the highest spring densities, followed by alfalfa (Fig. 2(b)). On the original scale, model 6 predicted autumn vole density in alfalfa crop to be about 1.5 times higher than in clover and more than twice as high as in alfalfa–grass mixtures. Third, the positive relationship between vole density and field size was further modified by altitude. This effect was less consistent and crop‐specific, increasing densities only in clover (model 7; Supporting Information, Fig. S1). By contrast, vole densities in alfalfa and alfalfa–grass mixtures were unaffected by elevation or slightly decreased.

**Table 1:** Comparison of linear mixed models using the Akaike Information Criterion (AICc) and the difference in AICc from the best model (*∆*AICc)

No.	Model structure	AICc	*Δ*AICc
1	Intercept only	10 862.3	48.0
2	Field size	10 828.4	14.1
3	Field size + Season	10 828.0	13.7
4	Field size + Season + Crop	10 828.0	13.7
**5**	**Field size + Season + Crop + Season × Crop**	**10 815.7**	**1.4**
**6**	**Field size + Season + Crop + Season × Crop + Altitude**	**10 814.3**	**0.0**
**7**	**Field size + Season + Crop + Season × Crop + Altitude**	**10 815.4**	**1.1**
	**+ Altitude × Crop**		
**8**	Field size + Season + Crop + Season × Crop + Altitude	10 817.7	3.4
	+ Field size × Crop + Field size × Altitude + Altitude × Crop		
**9**	**Field size + Season + Crop + Season × Crop +**	**10 815.2**	**0.9**
	**Field size × Crop + Field size × Season**		

The four best‐supported models are in shown bold.

## 4 DISCUSSION

Our results provide the first empirical evidence that vole densities increase with field size, although this relationship is strongly nonlinear and largely confined to fields smaller than 20 ha. We also found that vole densities differ among forage crops, with alfalfa supporting the highest autumnal populations, followed by clover and alfalfa–grass mixtures. These findings contribute to ecological theories on the abundance and population dynamics of voles and carry important implications for managing common vole populations in central European farmland.

Our study demonstrates that the size of the field planted with a preferred crop can significantly influence the abundance of a key herbivore and an important agricultural pest, providing support for earlier landscape studies.^20^, ^21^ Notably, this is the first documented case of a vole species exhibiting a positive density–area relationship, contrasting with earlier findings that reported no relationship (e.g. *Microtus agrestis*, *Clethrionomys glareolus*)^31^ or even negative relationships (e.g. *Microtus pennsylvanicus*, *Microtus ochrogaster*).^32^, ^33^ This pattern also contradicts prevailing theoretical expectations for ground‐dwelling dispersers, which predict lower densities in larger patches. Because an animal's ability to locate and reach a habitat patch scales with the patch's linear dimension rather than its area, larger patches are expected to receive fewer immigrants per unit area.^12^ This geometric constraint suggests that immigration alone cannot account for the observed increase in density with patch size. Additional demographic or behavioural processes must therefore contribute to the positive density–area relationship observed in the common vole.

We suggest that higher population densities in larger fields result from a greater likelihood that newly born individuals remain in the natal field rather than emigrating, thereby compensating for lower immigration rates per unit area. This mechanism is particularly plausible for fast‐breeding and highly social microtines such as the common vole. Under this hypothesis, populations in small patches are expected to exhibit higher emigration rates than those in larger patches. Support for this idea comes from experimental studies on emigration–immigration dynamics in patchy populations of the root vole (*Alexandromys oeconomus*),^34^ which indicate similar patterns.

Two additional variables affected vole population density: crop type and altitude. Although it has long been known that alfalfa (*Medicago sativa*) provides a high‐quality, protein‐rich food source for the common vole and supports high population densities,^35^, ^36^ our data offer more detailed insights into long‐term differences among forage crops. Specifically, we show that vole densities in autumn are highest in alfalfa crops, followed by clover and alfalfa–grass mixtures. This pattern aligns with previous research demonstrating that herbs, particularly legumes, constitute the primary food resource of the common vole.^37^ At the same time, our findings make it clear that although alfalfa is widely recognised for its agronomic and ecological benefits in farmland systems,^38^ it also supports higher vole abundances, which may increase the management costs associated with rodent damage. The observed positive effect of altitude on vole densities in clover fields appears to be novel; however, because of the limited data available, we cannot currently offer a plausible explanation for this pattern.

In recent years, the Czech government has responded to environmental concerns by implementing agri‐environmental subsidy programmes aimed at restoring landscape heterogeneity. A key condition of these schemes is the reduction of field size to below 30 ha, based on the premise that smaller fields with higher edge densities may support greater biodiversity and help mitigate pest outbreaks. However, the effectiveness of such measures remains largely untested in small mammal populations. In this context, our findings are highly relevant. We provide empirical evidence that reducing field size can indeed lower population densities of the common vole and, consequently, reduce crop damage. Because the relationship is noisy, however, its value lies chiefly in vole management at the national scale. For instance, reducing the current average field size from 18 to 13 ha (a 5‐ha decrease) may lower vole densities by ~8%. Because of the nonlinearity of the density–area relationship, the same reduction from 10 ha to 5 ha would result in an ~15% decline. Thus, the effect of such measures is expected to be most pronounced in smaller fields. However, because of substantial uncertainty in predicted local reductions of vole density for any given change in field size, the back‐transformed effect sizes are best interpreted as indicative of a nationwide tendency rather than precise local forecasts.

Our study presents the first empirical evidence that field size, a key aspect of landscape structure in farmland, influences the population density of the common vole in central European agriculture. By documenting a positive density–area relationship in a ground‐dwelling rodent, our findings challenge prevailing theoretical expectations and existing empirical studies, which have reported either no response or negative responses in voles. Given the economic and ecological significance of common vole population outbreaks, these results have practical implications: reducing field size may not only foster more resilient, heterogeneity‐rich agroecosystems, but also serve as a structural tool for mitigating pest pressure. Future research should investigate the behavioural mechanisms underlying vole responses to patch configuration and assess whether strategic landscape design can contribute to sustainable pest management in multifunctional agricultural landscapes.

## CONFLICT OF INTEREST

The authors report no conflict of interest.

## AUTHOR CONTRIBUTIONS

ET, RA, MB, MF and Jan Suchomel conceived the ideas and designed the study. ET collected and analyzed the data. All authors contributed to the final draft of the manuscript.’

## Supporting information


**Figure S1.** The interactive effect of altitude and crop types on vole population density as predicted by Model 7.

## Data Availability

The data that support the findings of this study are available on request from the corresponding author. The data are not publicly available due to privacy or ethical restrictions.
