# Supplementary material for: Field size as a determinant of common vole population density
Source: Pest Manag Sci. 2025 Nov 28;82(3):2740–5. doi: 10.1002/ps.70410 (PMC12886176; doi:10.1002/ps.70410)
Supplement: Supplementary file 1 — Figure S1. The interactive effect of altitude and crop types on vole population density as predicted by Model 7. [file PS-82-2740-s001.docx]

# Supplementary information: Data analysis

When analysing variation in vole population densities, we ended up with four best-supported linear mixed models: models 5, 6, 7 and 9 as reported in Table 1. Here, we provide detailed descriptive information on the statistical performance of these models as reported by the lme4 package in R. The function r.squaredGLMM from the MuMIn package was used to calculate pseudo-coefficients of determination.

## Model 6

Linear mixed model fit by maximum likelihood ['lmerMod']

Formula: index.bc ~ logfieldsize + season + crop + season:crop + altitude + (1 | year/season/district/crop) + (1 | fieldID)

Data: area

AIC BIC logLik -2*log(L) df.resid

10814.1 10895.8 -5393.1 10786.1 2517

Scaled residuals:

Min 1Q Median 3Q Max

-3.4858 -0.4965 0.0387 0.5647 2.7404

R^2^m: 0.057 R^2^c: 0.65

Random effects:

Groups Name Variance Std.Dev.

fieldID (Intercept) 1.5652 1.2511

crop:district:season:year (Intercept) 0.5998 0.7745

district:season:year (Intercept) 0.5450 0.7382

season:year (Intercept) 0.7914 0.8896

year (Intercept) 0.8472 0.9205

Residual 2.5096 1.5842

Number of obs: 2531, groups: fieldID, 781; crop:district:season:year, 546; district:season:year, 309; season:year, 14; year, 7

Fixed effects:

Estimate Std. Error t value

(Intercept) 5.42969 0.51972 10.447

logfieldsize 0.30212 0.05128 5.892

season.spring -0.23598 0.40377 -0.584

cropalfalfa 0.49925 0.22263 2.242

cropclo 0.09982 0.29824 0.335

altitude 0.12336 0.06668 1.850

season.sp:cropalfalfa 0.96874 0.23469 4.128

season.sp:cropclo 0.75274 0.30221 2.491

Correlation of Fixed Effects:

(Intr) lgfisi sesn.s crplf crpcl altitd ssn.s:crop

Logfldsize -0.175

season.s 0.014 -0.008

cropalflf -0.367 -0.155 -0.045

cropaclo -0.152 -0.157 -0.032 0.665

altitude -0.392 0.008 -0.007 0.036 -0.292

ssn.s:cropa -0.043 0.015 -0.505 0.076 0.058 0.009

ssn.s:cropc -0.027 0.018 -0.380 0.049 0.084 0.001 0.652

## Model 5

Linear mixed model fit by maximum likelihood ['lmerMod']

Formula: index.bc ~ logvymera + season + crop + season:crop + (1 | year/season/district/crop) + (1 | fieldID)

Data: area

AIC BIC logLik -2*log(L) df.resid

10815.5 10891.4 -5394.8 10789.5 2518

Scaled residuals:

Min 1Q Median 3Q Max

-3.4722 -0.4981 0.0329 0.5645 2.7502

R^2^m: 0.055 R^2^c: 0.65

Random effects:

Groups Name Variance Std.Dev.

katastr (Intercept) 1.5812 1.2575

plodina:district:season:rok (Intercept) 0.6068 0.7790

district:season:rok (Intercept) 0.5328 0.7299

season:rok (Intercept) 0.7883 0.8879

rok (Intercept) 0.8456 0.9196

Residual 2.5094 1.5841

Number of obs: 2531, groups: fieldID, 781; crop:district:season:year, 546; district:season:year, 309; season:year, 14; year, 7

Fixed effects:

Estimate Std. Error t value

(Intercept) 5.80714 0.47792 12.151

logfieldsize 0.30108 0.05143 5.854

season.s -0.23163 0.40323 -0.574

cropalfalfa 0.48434 0.22289 2.173

cropclo 0.26254 0.28575 0.919

season.s:cropalfalfa 0.96570 0.23495 4.110

season.s:cropclo 0.75156 0.30257 2.484

## Model 7

Linear mixed model fit by maximum likelihood ['lmerMod']

Formula: index.bc ~ logfieldsize + season + crop + season:crop + altitude + altitude:crop + (1 | year/season/district/crop) + (1 | fieldID)

Data: area

AIC BIC logLik -2*log(L) df.resid

10815.2 10908.6 -5391.6 10783.2 2515

Scaled residuals:

Min 1Q Median 3Q Max

-3.4769 -0.5059 0.0371 0.5670 2.7439

R^2^m: 0.058 R^2^c: 0.65

Random effects:

Groups Name Variance Std.Dev.

fieldID (Intercept) 1.5612 1.2495

crop:district:season:year (Intercept) 0.6154 0.7845

district:season:year (Intercept) 0.5148 0.7175

season:year (Intercept) 0.7899 0.8887

year (Intercept) 0.8476 0.9207

Residual 2.5102 1.5844

Number of obs: 2531, groups: fieldID, 781; crop:district:season:year, 546; district:season:year, 309; season:year, 14; rok, 7

Fixed effects:

Estimate Std. Error t value

(Intercept) 5.68863 0.69990 8.128

logfieldsize 0.30307 0.05132 5.906

season.s -0.23181 0.40380 -0.574

cropalfalfa 0.39171 0.56394 0.695

cropclo -1.07563 0.83889 -1.282

altitude 0.03953 0.16215 0.244

season.s:cropalfalfa 0.96596 0.23572 4.098

season.s:cropclo 0.73199 0.30341 2.413

cropalfalfa:altitude 0.03011 0.16223 0.186

cropclo:altitude 0.28194 0.20547 1.372

## Model 9

Linear mixed model fit by maximum likelihood ['lmerMod']

Formula: index.bc ~ logfieldsize + season + crop + season:logfieldsize + crop:logfieldsize + season:crop + (1 | year/season/district/crop) +

(1 | fieldID)

Data: area

AIC BIC logLik -2*log(L) df.resid

10814.9 10908.3 -5391.5 10782.9 2515

Scaled residuals:

Min 1Q Median 3Q Max

-3.4868 -0.4962 0.0406 0.5647 2.7644

R^2^m: 0.058 R^2^c: 0.66

Random effects:

Groups Name Variance Std.Dev.

fieldID (Intercept) 1.5687 1.2525

crop:district:season:year (Intercept) 0.5928 0.7699

district:season:year (Intercept) 0.5550 0.7450

season:year (Intercept) 0.7896 0.8886

year (Intercept) 0.8389 0.9159

Residual 2.5036 1.5823

Number of obs: 2531, groups: fieldID, 781; crop:district:season:year, 546; district:season:year, 309; season:year, 14; year, 7

Fixed effects:

Estimate Std. Error t value

(Intercept) 5.47910 0.51896 10.558

logfieldsize 0.56435 0.17738 3.182

season.s -0.35525 0.40671 -0.873

cropalfalfa 0.84605 0.31938 2.649

cropclo 0.74282 0.58214 1.276

logfieldsize:season.s 0.08596 0.04191 2.051

logfieldsize:cropalfalfa -0.27651 0.17928 -1.542

logfieldsize:cropclo -0.32105 0.24953 -1.287

season.s:cropalfalfa 0.89068 0.23883 3.729

season.s:cropclo 0.65486 0.30730 2.131


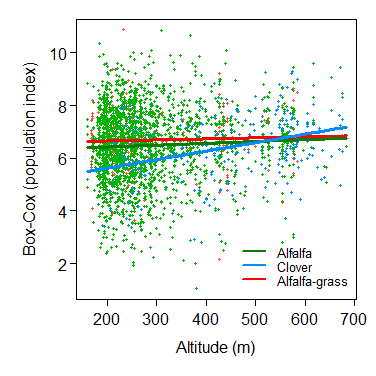


**Fig S1** The interactive effect of altitude and crop types on vole population density as predicted by Model 7.
